# Precision computerised cognitive behavioural therapy (cCBT) intervention for adolescents with depression (SPARX-UK): protocol for the process evaluation of a pilot randomised controlled feasibility trial

**DOI:** 10.1136/bmjopen-2024-092483

**Published:** 2025-08-05

**Authors:** Kareem Khan, Camilla May Babbage, Kirsty Sprange, Charlotte Lucy Hall, Adam Parker, Chris Greenhalgh, Matthew Jeffery, Mathijs Lucassen, Sally Merry, Vibhore Prasad, Karolina Stasiak, Boliang Guo, Christopher R Tench, Hannah Wright, Paul Stallard, Chris Hollis, on behalf of Digital Youth, Chris Hollis

**Affiliations:** 1 NIHR MindTech Medtech Co-operative, Institute of Mental Health, University of Nottingham, Nottingham, UK; 2 School of Applied Social Sciences, De Montfort University, Leicester, UK; 3 Mental Health & Clinical Neurosciences, School of Medicine, University of Nottingham, Nottingham, UK; 4 Nottingham Clinical Trials Research Unit, University of Nottingham, Nottingham, UK; 5 NIHR Nottingham Biomedical Research Centre, Nottingham, UK; 6 School of Computer Science, University of Nottingham, Nottingham, UK; 7 Single Point of Access, Oxfordshire Child and Adolescent Mental Health Service, Oxford Health NHS Foundation Trust, Oxford, UK; 8 School of Medicine, University of Auckland, Auckland, New Zealand; 9 School of Health and Medical Sciences, City St George’s, University of London, London, UK; 10 King’s College London, London, UK; 11 Lifespan and Population Health, School of Medicine, University of Nottingham, Nottingham, UK; 12 NIHR Applied Research Collaboration East Midlands, Nottingham, UK; 13 Department for Health, University of Bath, Bath, UK

**Keywords:** Adolescents, Depression & mood disorders, MENTAL HEALTH, Digital Technology, Protocols & guidelines

## Abstract

**Introduction:**

While digital technologies can increase the availability and access to evidence-based interventions, little is known about how users engage with them and the mechanisms associated with effective outcomes. Process evaluations are an important component in understanding the aforementioned factors. The ‘SPARX-UK’ study is a randomised controlled pilot and feasibility trial evaluating personalised human-supported (from an ‘eCoach’) vs a self-directed computerised cognitive behavioural therapy intervention (cCBT), called SPARX (Smart, Positive, Active, Realistic, X-factor thoughts), aimed at adolescents with mild to moderate depression. We are comparing supported vs self-directed delivery of SPARX to establish which format should be used in a proposed definitive trial of SPARX. The control is a waitlist group. We will conduct a process evaluation alongside the trial to determine how the intervention is implemented and provide context for interpreting the feasibility trial outcomes. We will also look at the acceptability of SPARX and how users engage with the intervention. This protocol paper describes the rationale, aims and methodology of the SPARX-UK trial process evaluation.

**Methods and analysis:**

The process evaluation will use a mixed-methods design following the UK Medical Research Council’s 2015 guidelines, comprising quantitative and qualitative data collection. This will include analysing data usage of participants in the intervention arms; purposively sampled, semi-structured interviews of adolescents, parents/guardians, eCoaches and clinicians/practitioners from the SPARX-UK trial; and analysis of qualitative comments from a survey from those who dropped out early from the trial. Quantitative data will be analysed descriptively. We will use thematic analysis in a framework approach to analyse qualitative data. Quantitative and qualitative data will be mixed and integrated to provide an understanding of how the intervention was implemented and how adolescents interacted with the intervention. This process evaluation will explore the experiences of adolescent participants, parents/guardians, eCoaches and clinicians/practitioners in relation to a complex digital intervention.

**Ethics:**

Ethical approval was granted by the National Health Service (NHS) Health Research Authority South West - Cornwall & Plymouth Research Ethics Committee (Ethics Ref: 22/SW/0149).

**Dissemination:**

Contextualising how the intervention was implemented, and the variations in uptake and engagement, will help us to understand the trial findings in greater depth. The findings from this process evaluation will also inform the decision about whether and how to proceed with a full randomised controlled trial, as well as the development of more effective interventions which can be personalised more precisely via varying levels of human support. We plan to publish the findings of the process evaluation and the wider project in peer-reviewed journals, as well as disseminate via academic conferences.

**Trial registration number:**

ISRCTN: ISRCTN15124804. Registered on 16 January 2023, https://www.isrctn.com/ISRCTN15124804.

**Strengths and limitations of this study**

- Framework analysis allows for in-depth and rigorous qualitative analysis using a transparent method.
- The process evaluation will use robust quantitative and qualitative methods grounded within a theoretically informed logic model.
- Amalgamation of data sources will maximise credibility and validity.
- The evaluation may be limited or skewed towards more positive experiences of the intervention; however, a wider survey has been developed to capture those that do not engage well with the intervention or withdraw from the study.

## Introduction

 Depression is characterised by a persistent low mood, lack of energy, anhedonia, poor concentration and a myriad of other symptoms affecting quality of life, and it is also associated with suicide.^1^ Depression has been recognised as one of the world’s leading health problems affecting up to 3.8% of people globally.^2^ Adolescents (ie, those aged 10–19 years) are at a particularly vulnerable stage in their lives and rates of depression among this demographic are increasing,^2 3^ with associated costs to individuals and society.^4^ For adolescents, the National Institute for Health and Care Excellence (NICE) recommends cognitive behavioural therapy (CBT) as a first-line treatment for depression.^5^ CBT centres around challenging unhelpful thoughts and focusing on changing behaviours, while also encouraging cognitive restructuring, problem-solving, behavioural activation and homework tasks.^6^

CBT has a large evidence base,^7 8^ yet access to evidence-based treatment such as CBT is lowest among adolescents^9^ with only 25% of those requiring it receiving appropriate treatments.^10^ Reasons for poor access to treatments include a lack of resources from services including availability of therapists and long waitlists,^11^ or barriers experienced by adolescents such as stigma and embarrassment.^12^ Due to their affinity for technology, a promising development that may benefit adolescents is online or digital health interventions (DHIs). Computerised CBT (cCBT) is one such DHI that is effective for adults and adolescents with depression.^13^,^15^ Despite the efficacy of DHIs, such as cCBT, there have been several issues with low engagement and adherence with unsupported interventions. For example, a study evaluating a self-directed DHI called MoodGYM in secondary schools found that only 8.5% of participants logged on to use the intervention.^16^ However, it is widely known that a commonly purported facilitator that improves engagement and adherence with DHIs is human support.^17^,^19^

In recent years, cCBT has become more interactive and aesthetically attractive to young people with the advent of ‘serious games’.^20^ Serious games are “games that do not have entertainment, enjoyment, or fun as their primary purpose.”^21^ An example of a serious game is SPARX (Smart, Positive, Active, Realistic, X-factor thoughts). SPARX was originally developed in New Zealand as a self-help cCBT intervention. In a non-inferiority trial, where SPARX was compared with treatment as usual (TAU) among adolescents seeking help for their depressive symptoms in New Zealand, SPARX was not inferior to TAU (face-to-face therapy), with a significant post-intervention mean reduction on the primary outcome measure of 10.32 for SPARX compared with 7.59 for TAU.^22^ These improvements were maintained at 3-month follow-up. Fleming *et al*^23^ conducted a pragmatic trial comparing SPARX to a waitlist control and found there were statistically significant reductions in depression (–4.6 vs +3.2) and anxiety symptoms (–14.7 vs –1.1) among young people excluded from mainstream education in New Zealand. Also in New Zealand, Lucassen *et al*^24^ found a statistically significant decrease in depressive symptoms in sexual minority (eg, lesbian, gay and bisexual) adolescents using the Rainbow version of SPARX from pre-intervention to post-intervention (effect size d=1.01 for this open trial), which were maintained at 3-month follow-up. A depression prevention trial carried out in Australia found that participants in the SPARX-R (a resilience-focused version of the intervention^25^) condition showed significantly reduced symptoms of depression relative to the control group at post-intervention (d=0.29) and 6 months post-baseline (d=0.21).^26^ Indeed, a systematic review of digitally delivered studies favoured SPARX as the most promising DHI for young people with depression and anxiety.^27^ However, there have been studies inside^28^ and outside of Australasia which have failed to show efficacy^29 30^. As the literature suggests, interventions are context-dependent, and efficacy in one country does not necessarily translate to efficacy in other countries. Thus, there is a need to further evaluate SPARX outside of its country of origin.

Before any new DHI is introduced, clinicians, patients and commissioners need robust evidence that demonstrates effectiveness as well as acceptability, adherence and use of the intervention data. This includes any apparent impact of the digital divide on health inequalities and on the resources and activities required to achieve effective implementation. Little is known about how, and for whom in particular DHIs work, what makes them effective in one context and not in another and a full understanding of the barriers to effective implementation is still unknown.^31 32^ To support the development of complex interventions, such as DHIs, the UK Medical Research Council (MRC) stipulates that for pilot and feasibility trials in particular, process evaluations are essential to determine the decision to proceed to a full randomised controlled trial (RCT) and to optimise the intervention going forward.^33^ Process evaluations can therefore aid the interpretation and understanding of trial outcomes, as well as inform future refinements of the intervention under study. In order to assess the specific components of a process evaluation, namely the quality of implementation (fidelity), dose, reach and adaptations, analyse causal mechanisms and identify any contextual factors, we will follow the MRC guidelines on conducting process evaluations.^34^

### SPARX-UK trial overview

As reported in the study protocol^35^ (26/03/2024; version 1.0), the SPARX-UK trial is a three-armed, single-blind, pilot randomised controlled feasibility trial. Originally evaluated by Merry *et al*,^22^ we aim to evaluate the feasibility of the SPARX intervention in the UK with adolescent patients who have mild to moderate depression. Participants will be randomised into one of three groups: supported SPARX, self-directed unsupported SPARX and a waitlist control group (ie, treatment as usual). The former will receive 8–10 weeks of seven supported modules (levels) of CBT with access to an ‘eCoach’ (assistant psychologist) to offer personalised interventional support, but no additional therapeutic input (ie, no therapeutic techniques will be specified beyond that outlined in SPARX). The comparator is self-directed access to SPARX with no additional support. We chose a waitlist as the control group because we have two active groups; thus, we are not only comparing to the waitlist group. Moreover, the extra support given to young people as part of being in a study can be beneficial, which was shown in the Dutch study of SPARX^30^ whereby those in the monitored controlled group also saw improvements. Both active groups will access SPARX via a secured app or internet browser. Participants must be aged 11–19 years old with symptoms of depression, able to provide written consent or, if under age 16, written assent and parental/guardian consent, with access to a computer and smartphone, and be able to read and write in English. Patients will be excluded where there are clinical concerns that depression is too severe and self-harm/suicidal risk is too high, intellectual disability or physical limitations precluding the use of SPARX, received (in the past 3 months) or currently receiving treatment with CBT/interpersonal therapy, has another major mental health disorder (eg, psychosis, eating disorder) where the primary focus was not depression and safeguarding concerns that are not currently being managed (ie, the young person is the subject of a safeguarding investigation). Participants will be followed up mid-treatment (4 weeks post-randomisation) and at 8–10 weeks post-randomisation (primary endpoint).

SPARX features seven modules employing CBT principles whereby participants undertake a series of challenges to restore balance in a fantasy world dominated by GNATs (Gloomy Negative Automatic Thoughts). The game consists of seven modules to be completed sequentially, lasting about 30 minutes each. Throughout the 8–10 weeks, those in the supported arm will have access to an eCoach. The role of the eCoach is to personalise the level of support, encourage participants to engage with the treatment content and its homework assignments, as well as answer any queries that arise. Parents/guardians in the supported and unsupported SPARX groups are also given a manual that contains information including what the key learning objectives are for each module, what questions the parent/guardian could pose to their child after each module and what skills their child may be able to practise after each module. The target sample size for the SPARX-UK trial is N=120 (n=40 in the supported SPARX arm, n=40 in the self-directed unsupported SPARX arm and n=40 in the waitlist control arm). A sample size of 40 per arm should be sufficient to avoid under or overpowering the main study, while not making the pilot study excessively large.^36^

### Aims of the process evaluation

The SPARX-UK process evaluation aims to follow MRC guidelines^34^ to understand both the functioning of the trial processes and the intervention itself. This understanding is crucial to the development and decision to proceed to a full RCT. This will be achieved by seeking to understand potential relationships between factors obtained from trial data. This will be completed by exploring the fidelity of intervention delivery, acceptability of and satisfaction with the intervention, and reasons for the observed variation in uptake, adherence and engagement. The resources and implementation processes required for a full RCT will also be considered. Specific research questions include:

#### Fidelity

1. To what extent is the intervention delivered as intended in both the supported and self-directed formats?
2. What are the patterns of engagement and adherence across both SPARX conditions?
3. How do participants perceive the accessibility, usability and acceptability of the intervention?

#### Mechanisms of impact

1. What are the key therapeutic mechanisms (eg, cognitive restructuring, problem-solving, behavioural activation) that are thought to contribute to symptom reduction?
2. How does eCoach support appear to influence engagement and outcomes in the supported SPARX condition?
3. What role does gamification seem to play in sustaining engagement and adherence?

#### Context

1. What participant characteristics (eg, baseline symptom severity, age, gender) likely moderate engagement and intervention outcomes?
2. What external factors (eg, parental support) are identified as influencing adherence and effectiveness?
3. How do participants in different demographic groups (eg, age, gender) experience and engage with the intervention?

**Figure 1:**
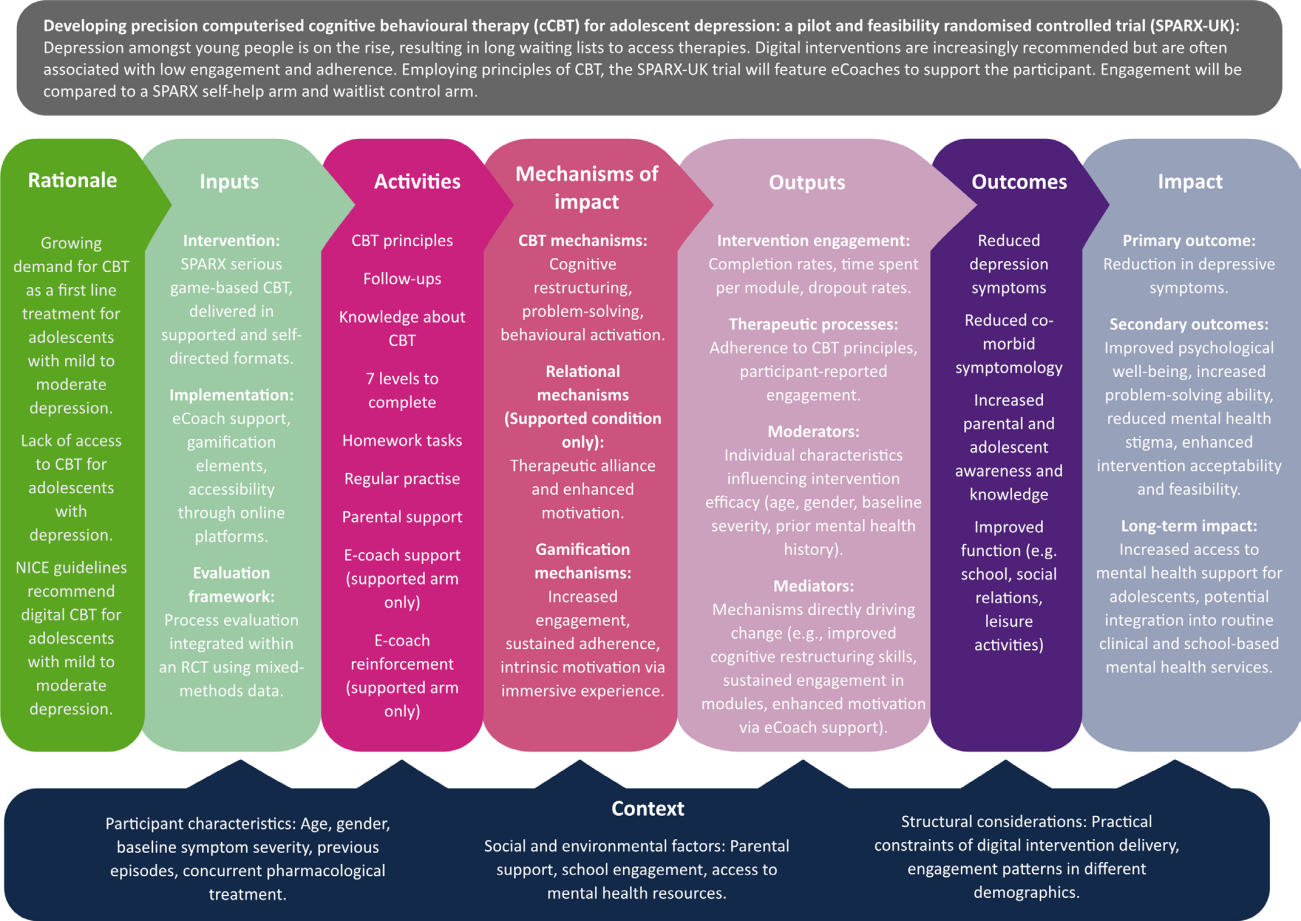
Logic model for SPARX-UK (Smart, Positive, Active, Realistic, X-factor thoughts).

MRC guidance on the development and evaluation of complex interventions notes that identifying and developing a theoretical understanding of the likely process of change is an early key task for developing a complex intervention or evaluating one that has already been developed. These guidelines stipulate an important component of a process evaluation is to outline the processes of the intervention and the outcomes it aims to achieve through a logic model. The logic model for the study is shown in figure 1.

## Methods and analysis

### Overall design

As there are few published protocols of process evaluations, the research team’s previous work on the Online Remote Behavioural Intervention for Tics (ORBIT) trial was used as a guide for the design of this evaluation.^37^ The overall design of the SPARX-UK process evaluation is a mixed-methods study using purposively sampled qualitative and quantitative data from the trial (please see the trial protocol for study information related to the trial^35^). This will involve semi-structured interviews with adolescents, parents/guardians, eCoaches and clinicians/practitioners, analyses of online survey responses from participants together with data from the online platform, such as total eCoach time (if in the supported arm), number of modules completed and number of logins.^34^ Data collection began in March 2023 and ends in June 2025.

In online supplemental file 1, a populated Standard Protocol Items: Recommendations for Interventional Trials (SPIRIT) checklist is provided.^38^

### Patient and public involvement and engagement

The current project sits within a programme of work with its own Patient and Public Involvement and Engagement (PPIE) group of young people with lived experience of accessing services for depression/mental health conditions, called Sprouting Minds. The SPARX-UK PPIE group consists of three young people and one parent with lived experience of personal or children’s mental ill-health. They have been consulted at every stage of the research design and have provided advice and guidance on which outcome measures to use, and, for the process evaluation in particular, which topics to include in qualitative interviews. The PPIE group will be invited to support the analysis of the qualitative interviews which will include attending training in qualitative analysis and will be offered opportunities to coproduce codes and themes during analysis. Furthermore, feedback from PPIE will be used to support the dissemination of the research and development of future grants.

### Qualitative data collection

Qualitative data will be collected by interviewing adolescent participants and their parents/guardians in the SPARX-UK trial, eCoaches and referring clinicians/practitioners. All interviews will be conducted either by telephone or by videoconferencing. We will also conduct a survey to capture views from those who dropped out of the trial/intervention early (see online supplemental file 2). This is to ensure that the process evaluation is not overly skewed towards positive experiences of the trial/intervention and that we gain views from those who potentially may not have had a positive experience. Finally, any notes completed by young people in the online journal integrated within the SPARX intervention will be downloaded and analysed.

#### Sampling and recruitment for adolescent and parent/guardian interviews

Researchers will inform the parent/guardian and adolescent at the primary endpoint that they may be invited to interview but their participation is optional. Only those who provided explicit written consent will be approached to participate in an interview for the SPARX-UK trial (at the time of consenting to the study) and, for a child under 16, assent was obtained with parental/guardian consent (see online supplemental file 3). If the parent/adolescent agrees, an unblinded interviewer not involved in trial duties will contact the parent/guardian to arrange a suitable day and time for an interview. All participants who received SPARX (in the unsupported or supported arms) will be approached to be interviewed and we anticipate that this sampling strategy will result in sufficient heterogeneity to provide examples of both poor and good adoption, delivery and maintenance, and will allow us to identify barriers and facilitators to implementation. It will also allow us to generate hypotheses about factors that may be associated with differing outcomes for participants.

The target for interviews will be approximately 20% of the sample for each arm, for parents/guardians of adolescents and adolescents. This sample size was determined a priori based on the model of information power, whereby we aim to achieve both breadth and depth of views.^39^ This will also ensure that data will reach a level of saturation^40^ and enable a diversity of views.

All interview schedules were drafted and underwent revision by members of the study team and our PPIE group. Questions for adolescent participants include: (a) how they heard about the trial; (b) why they took part; (c) their initial expectations; (d) their views of the content, structure and the different levels of SPARX; (e) what impact the intervention had, if any, on their depression; (f) what they found most and least helpful; (g) barriers to participation; (h) how they felt about communicating with their eCoach (if in the supported arm); (i) if they would alter anything about the intervention; (j) their recommendations for improvement of the intervention and their overall experience of participating in the trial. The questions for parents/guardians are similar, with the added aspect of how they supported their child throughout the trial.

We will carry out interviews with adolescents and parents/guardians following the completion of the intervention at the primary endpoint assessment in the main trial. Interviews will be conducted by a trained researcher and we will use Microsoft Teams or telephone to record interviews depending on participant preference. All interviews will be transcribed verbatim using tools provided by videoconferencing platforms or by the automatic transcription service at the University of Nottingham.

#### Sampling and recruitment for eCoaches and clinician/practitioner interviews

Questions for eCoaches include (a) their clinical or research experience prior to their role in the trial; (b) how they found out about SPARX-UK and why they got involved; (c) what specific skills they felt were needed to support adolescents; (d) any support from supervisors required or any training needs identified; (e) how they managed SPARX-UK around other commitments; (f) if the intervention is being delivered as planned; (g) their experiences of interacting with participants; (h) their views on the trial arms; (i) and their recommendations for future use. All eCoaches with experience of supporting at least three participants as eCoach will be invited to interview. This will ensure that we interview eCoaches with sufficient experience of supporting participants using SPARX.

Clinicians/practitioners refer to any healthcare professional who is responsible for referring participants to the SPARX-UK trial or supervising those who refer into the trial, and who are not explicitly involved in the trial. The clinician/practitioner interview schedule questions aim at eliciting information on their clinical background and experience, any research experience they may have had and how they got involved in the SPARX-UK trial and why. Questions will explore their experience of recruiting for the trial including factors that affected recruitment, any resources that were or would have been required to recruit into the trial and how the National Health Service (NHS) could incorporate the intervention into everyday practice. Clinicians from across all recruiting sites for SPARX-UK will be invited to interview, with a target of n=5 for clinician interviews. This will ensure that data reach a level of saturation.

All interview schedules are presented in online supplemental file 4.

### Quantitative data collection

#### Process data

We will collect and record online data from participants throughout the trial. This includes the following measures: total time spent with eCoach, the total number of participant logins, time spent on each module and the total number of modules completed. This data will be amalgamated and entered into a centralised online database whereby the researcher will then extract this data for analysis as part of the process evaluation.

### Data analysis

#### Qualitative data analysis

Qualitative data will be exported and analysed using a mix of spreadsheet software to support collaborative analysis and qualitative analysis software with QSR International’s NVivo 14 Software.^41^ Transcripts will be checked for accuracy against the recordings with any corrections made as appropriate. Before the transcripts are imported into NVivo 14, the process evaluation researcher will remove any reference to places, clinicians, eCoaches and/or family members that may reveal participants’ identities, and all participants’ names will be anonymised.

As the process evaluation is a combination of exploration and description, thematic analysis will be used to identify, analyse and report patterns within the transcribed interviews.^42^ Thematic analysis is widely used within health research and is considered the most flexible qualitative analytical process.^42^ By this, we mean that thematic analysis can be applied across methodologies and epistemologies.^43^ More broadly, we will employ framework analysis,^44^ as it is most commonly used for the thematic analysis of large datasets of semi-structured interviews.^45^

Following Ritchie & Spencer’s^44^ five stages of framework analysis, we will complete the stages including familiarisation, identifying a thematic framework, indexing, charting, mapping and interpretation. The familiarisation stage includes the researcher familiarising themself with the data through listening and watching the interviews, re-reading the transcriptions and studying any observational notes, and simultaneously listing key ideas or themes. Subsequently, analysis of the data will identify key issues, concepts, themes and subthemes, considering both a priori and emergent issues. The next stage includes systematically applying the thematic framework to each interview through coding transcripts and indexing these into framework categories. Working through each framework category, the indexed data will become summarised and organised into a chart for each participant, using headings and subheadings. Together, the whole data set will be mapped and interpreted with its key characteristics. A different member of the team will double-code a subset of transcripts to identify patterns and themes relating to participants’, eCoaches’ and clinicians’ experiences. The data that have been charted will be annotated by team members independently, and back-and-forth discussions will take place to refine and amend the data ensuring that the process is iterative. Both researchers will assess how confident they feel that the interpretation is congruent and meaningful, ahead of reviewing any remaining interviews, to ensure acceptability in its understanding.

Moreover, at each stage of the analysis, our PPIE group will be consulted, and they will be trained and supported in the analysis of the qualitative data, including the development of codes and theming of the clusters. Any differences of opinion will be taken to research team meetings and discussed as a group, and where resolution cannot be found, differences will be reported.

#### Quantitative data analysis

Quantitative data will be exported and analysed in IBM (International Business Machines Corporation) SPSS Statistics (Version 29).^46^ Quantitative data from the online platform will be subject to descriptive statistical analysis with total numbers and percentages and mean with SD or median (range), if not normally distributed, being presented. This will provide information on intervention delivery, including the implementation of different components and fidelity. Independent samples χ² and t-tests will be calculated to explore any significant differences between groups. For data not normally distributed, non-parametric alternatives will be used (ie, Kruskal–Wallis H and Mann–Whitney U tests). Contextual variables including age, gender, baseline symptom severity and concurrent pharmacological treatment will be examined in multiple linear regression models. All statistical analyses will use a significance level of p<0.05. Given that our feasibility study is not powered for definitive between-group comparisons of efficacy but aims primarily to assess feasibility, acceptability and mechanisms of engagement, process evaluation quantitative analyses will mainly be exploratory.

#### Mixed methods analysis

We will adopt a convergent mixed-methods design,^47^ where qualitative and quantitative data are analysed separately and then merged for interpretation. To link the data, we will use a group-level integration strategy, meaning that we will match qualitative interview responses with the corresponding quantitative trial data for the same groups (ie, supported vs self-directed SPARX). This allows us to explore and compare how fidelity, mechanisms and contextual influences relate to observed trial outcomes and implementation in the two groups. We will present these findings using joint displays, a recommended technique for visualising integrated mixed-methods results.^48^ We will give both qualitative and quantitative data equal importance, as both sets of data are central to addressing the research questions conceived by the process evaluation.

In online supplemental file 5, a Good Reporting of A Mixed Methods Study (GRAMMS)^49^ checklist has been provided.

#### Integration of findings

The process evaluation data will be analysed before knowing the main SPARX-UK pilot trial results with the two analyses being independent of each other. Following quantitative analysis of SPARX-UK trial data, qualitative data from the process evaluation can potentially be used to help explain the outcomes of the trial. Additional analyses can then be conducted to test hypotheses emanating from the integration of process evaluation data with trial outcomes, drawing together the findings to understand why the intervention worked (or not), the context and the implications for proceeding to a full RCT. By collecting data from a range of relevant stakeholders (eg, parents, adolescents, eCoaches and clinicians) and combining quantitative and qualitative data, we will gain a holistic understanding of the mechanisms underlying the impact and implementation of the intervention.

### Ethics and dissemination

The SPARX-UK trial obtained ethical approval from the NHS Health Research Authority South West - Cornwall & Plymouth Research Ethics Committee on 15 December 2022 (Ethics Ref: 22/SW/0149) and has been registered with the International Standard Randomised Controlled Trial Number Registry (ISRCTN; trial number 15124804). Full details of consent procedures and how we will manage safety (ie, monitoring suicidality and monitoring and reporting adverse events) are described in our published protocol paper.^35^ Findings will be published in open-access, peer-reviewed scientific journals and presented at conferences and seminars with the involvement of PPIE to support our dissemination processes.

## Discussion

This protocol outlines the rationale, design and methodology for the planned mixed-methods process evaluation of SPARX-UK, a serious game for adolescents with depression. The process evaluation is designed to explore the implementation of the online intervention and provide a holistic view of trial outcomes. By explicitly outlining our process evaluation methodology, guided by the MRC framework of complex intervention trials, this paper adds to the literature on process evaluation protocols using a mixed-methods design. Doing so will improve the integrity of this process evaluation and, as mentioned, there is growing emphasis on the importance of publishing process evaluation protocols in advance to improve overall trial quality and reporting.^50^ The combined qualitative and quantitative process evaluation data will support the homogenous interpretation of the main outcome data from the SPARX-UK trial. By providing an illumination of how and why SPARX was effective or not, the process evaluation will help elucidate a holistic view of the intervention. Moreover, understanding the implementation, mechanisms of impact and any contextual factors, this data will augment the dissemination plan and will aid in the decision to proceed to a full RCT.

## Supplementary material

10.1136/bmjopen-2024-092483 online supplemental file 1

10.1136/bmjopen-2024-092483 online supplemental file 2

10.1136/bmjopen-2024-092483 online supplemental file 3

10.1136/bmjopen-2024-092483 online supplemental file 4

10.1136/bmjopen-2024-092483 online supplemental file 5
