# Supplementary material for: Precision computerised cognitive behavioural therapy (cCBT) intervention for adolescents with depression (SPARX-UK): protocol for the process evaluation of a pilot randomised controlled feasibility trial
Source: BMJ Open. 2025 Aug 5;15(8):e092483. doi: 10.1136/bmjopen-2024-092483 (PMC12336577; doi:10.1136/bmjopen-2024-092483)
Supplement: online supplemental file 2 [file bmjopen-15-8-s002.docx]

# Participant Experience Questionnaire

Please help us improve SPARX by answering some questions about the treatment you received. We would like to know why you decided that SPARX perhaps was not suitable for you. We are interested in your honest opinions, whether they are positive or negative as this will help us with SPARX going forward.

Please answer all the questions. We also welcome your comments and suggestions.

1. **What was the main reason for you wanting to take part in the study?**

Improvement of my mood

My doctor suggested it might be helpful to me

Family/friend suggested it might be helpful

The research might help other people and improve health services

Other, please specify

1. **Can you tell us if any of the following were reasons for you no longer wanting to take part in the study? (Please tick as many that apply)**

I did not receive SPARX

I did not find SPARX helpful

I struggled with the technology aspect

I would have preferred to have face-to-face therapy

I do not think I need SPARX

Length of the study

My health is not very good now

Time commitments

Too many questionnaires to complete

Other commitments please specify

I struggled to communicate with the e-coach

Other, please specify

1. **Is there anything else you would like to add in relation to the study or SPARX?**

[Free comment box]
