# Supplementary material for: Precision computerised cognitive behavioural therapy (cCBT) intervention for adolescents with depression (SPARX-UK): protocol for the process evaluation of a pilot randomised controlled feasibility trial
Source: BMJ Open. 2025 Aug 5;15(8):e092483. doi: 10.1136/bmjopen-2024-092483 (PMC12336577; doi:10.1136/bmjopen-2024-092483)
Supplement: online supplemental file 3 [file bmjopen-15-8-s003.docx]

**
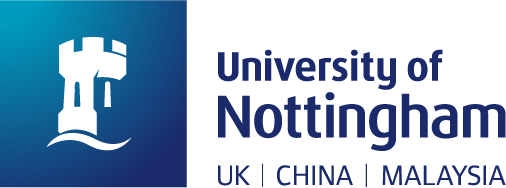
**

**ASSENT FORM FOR YOUNG PEOPLE UNDER 16 YEARS**

**For ONLINE**

(Final version 2.0: 27.10.22)

**Title of Study:** Developing precision computerised cognitive behavioural therapy (cCBT) for adolescent depression (SPARX-UK)

**REC reference:** 22/SW/0149

**IRAS Project ID:** 316644

This research has been funded by: Medical Research Council (Ref: MR/W002450/1)

Please click YES to each statement below to participate in the study. If there is any statement you don’t agree with, please exit the survey by closing this tab/browser window and you will not take part in the study.

1. I have read the information sheet version number [] dated [ ] for the SPARX-UK study. I have discussed it with my mum/dad/guardian and the researcher, and I have asked questions.

**YES**

1. I understand that I don’t have to take part and I can stop taking part any time. This is my choice, and no one will be upset with me if I stop.

**YES**

1. I understand that the SPARX-UK team may look at data collected in the study. This will be kept safe and only the research team will see my data.

**YES**

1. I understand that the research team will write a report about the project. My name will not be mentioned in any reports.

**YES**

1. I agree to my GP knowing that I am taking part in the SPARX-UK study.

**YES**

**OPTIONAL**

1. The researcher might ask me to take part in an interview about my experiences of the SPARX-UK trial. I do not have to take part. If I agree to take part, the interview will be audio recorded only and anonymous direct quotes from these interviews may be used in study reports but only the research team will know that I did the interview.

**YES**

1. I agree to take part in the SPARX-UK study.

**YES**

**Please type your name below (this will be taken as your signature to agree to take part in the study)**

Name Date

**CONSENT FORM FOR YOUNG PEOPLE 16 YEARS AND OVER**

**For ONLINE**

**(Final version 2.0: 27.10.22)**

**Title of Study:** Developing precision computerised cognitive behavioural therapy (cCBT) for adolescent depression (SPARX-UK)

**REC reference: 22/SW/0149**

**IRAS Project ID:** 316644

This research has been funded by: Medical Research Council (Ref: MR/W002450/1)

Please click YES to each statement below to participate in the study. If there is any statement you don’t agree with, please exit the survey by closing this tab/browser window and you will not take part in the study.

1. I confirm that I have read and understood the information sheet version number [] dated [] for the above study. I have had the opportunity to consider the information, ask questions and have had these answered satisfactorily.

**YES**

1. I understand that my participation is voluntary and that I am free to withdraw at any time without giving any reason, without my medical care or legal rights being affected. I understand that should I withdraw then the information collected so far cannot be erased and that this information may still be used in the project analysis.

**YES**

1. I understand that relevant sections of data collected during the study may be looked at by individuals from the SPARX-UK team, from regulatory authorities or from the NHS Trust, where it is relevant to my taking part in this research. I give permission for these individuals to have access to my records and to collect, store, analyse and publish information obtained from my participation in this study. I understand that my personal details will be kept confidential.

**YES**

1. I understand that the information collected about me will be used to support other research in the future and may be shared anonymously with other researchers.

**YES**

1. I agree to my GP being informed of my participation in the study.

**YES**

**OPTIONAL**

1. I understand that I may be asked to take part in research interviews, which will be audio recorded only and anonymous direct quotes from these interviews may be used in study reports.

**YES**

1. I agree to take part in the above study.

**YES**

**Please type your name below and provide an e-signature**

Name Date

e-signature:

**CONSENT FORM FOR PARENTS/GUARDIANS**

**For ONLINE**

**(Final version 2.0: 27.10.22)**

**Title of Study:** Developing precision computerised cognitive behavioural therapy (cCBT) for adolescent depression (SPARX-UK)

**REC reference: 22/SW/0149**

**IRAS Project ID:** 316644

This research has been funded by: Medical Research Council (Ref: MR/W002450/1)

Please click YES to each statement below to participate in the study. If there is any statement you don’t agree with, please exit the survey by closing this tab/browser window and you will not take part in the study.

1. I confirm that I have read and understood the information sheet version number [] dated [ ] for the above study. I have had the opportunity to consider the information, ask questions and have had these answered satisfactorily.

**YES**

1. I understand that mine and my child’s participation is voluntary and that I am free to withdraw at any time without giving any reason, without my or my child’s medical care or legal rights being affected. I understand that should I withdraw then the information collected so far cannot be erased and that this information may still be used in the project analysis.

**YES**

1. I understand that relevant sections of data collected about me and my child during the study may be looked at by individuals from the SPARX-UK team, from regulatory authorities or from the NHS Trust, where it is relevant to my taking part in this research. I give permission for these individuals to have access to these records and to collect, store, analyse and publish information obtained from my participation in this study. I understand that my personal details will be kept confidential.

**YES**

1. I understand that the information collected about me, and my child will be used to support other research in the future and may be shared anonymously with other researchers.

**YES**

1. I agree to my child’s General Practitioner being informed of our participation in the study.

**YES**

**OPTIONAL**

1. I understand that I/my child may be asked to take part in research interviews, which will be audio recorded only and anonymous direct quotes from these interviews may be used in study reports.

**YES**

1. I agree for me and my child (named below) to take part in the above study.

**YES**

**Please type yours and your child’s name below and provide an e-signature**

Name Date

Name of child

e-signature:

**CONSENT FORM FOR PARENTS/GUARDIANS only**

**For ONLINE**

**(Final version 2.0: 27.10.22)**

**Title of Study:** Developing precision computerised cognitive behavioural therapy (cCBT) for adolescent depression (SPARX-UK)

**REC reference: 22/SW/0149**

**IRAS Project ID: 316644**

This research has been funded by: Medical Research Council (Ref: MR/W002450/1)

Please click YES to each statement below to participate in the study. If there is any statement you don’t agree with, please exit the survey by closing this tab/browser window and you will not take part in the study.

1. I confirm that I have read and understood the information sheet version number [] dated [ ] for the above study. I have had the opportunity to consider the information, ask questions and have had these answered satisfactorily.

**YES**

1. I understand that my participation is voluntary and that I am free to withdraw at any time without giving any reason, without my medical care or legal rights being affected. I understand that should I withdraw then the information collected so far cannot be erased and that this information may still be used in the project analysis.

**YES**

1. I understand that data collected about me during the study may be looked at by individuals from the SPARX-UK team, from regulatory authorities or from the NHS Trust, where it is relevant to my taking part in this research. I give permission for these individuals to have access to these records and to collect, store, analyse and publish information obtained from my participation in this study. I understand that my personal details will be kept confidential.

**YES**

1. I understand that the information collected about me will be used to support other research in the future and may be shared anonymously with other researchers.

**YES**

**OPTIONAL**

1. I understand that I may be asked to take part in research interviews, which will be audio recorded only and anonymous direct quotes from these interviews may be used in study reports.

**YES**

1. I agree for me to take part in the above study.

**YES**

**Please type your name below and provide an e-signature**

Name Date

e-signature:

**CONSENT FORM FOR E-COACH**

**For ONLINE**

**(Final version 2.0: 27.10.22)**

**Title of Study:** Developing precision computerised cognitive behavioural therapy (cCBT) for adolescent depression (SPARX-UK)

**REC reference: 22/SW/0149**

**IRAS Project ID: 316644**

This research has been funded by: Medical Research Council (Ref: MR/W002450/1)

Please click YES to each statement below to participate in the study. If there is any statement you don’t agree with, please exit the survey by closing this tab/browser window and you will not take part in the study.

1. I confirm that I have read and understood the information sheet version number [] dated [ ] for the above study. I have had the opportunity to consider the information, ask questions and have had these answered satisfactorily.

**YES**

1. I understand that my participation is voluntary and that I am free to withdraw at any time without giving any reason, without my legal rights being affected. I understand that should I withdraw then the information collected so far cannot be erased and that this information may still be used in the project analysis.

**YES**

1. I understand that relevant sections of my data collected in the study may be looked at by authorised individuals from the University of Nottingham, the research group, and regulatory authorities where it is relevant to my taking part in this study. I give permission for these individuals to have access to these records and to collect, store, analyse and publish information obtained from my participation in this study. I understand that my personal details will be kept confidential.

**YES**

1. I understand that the interview will be audio recorded only and that anonymous direct quotes from the interview may be used in the study reports.

**YES**

5. I agree to take part in the above study.

**YES**

**Please type your name below (this will be taken as your signature to agree to take part in the study)**

Name Date

**CONSENT FORM FOR CLINICIANS**

**For ONLINE**

**(Final version 2.0: 27.10.22)**

**Title of Study:** Developing precision computerised cognitive behavioural therapy (cCBT) for adolescent depression (SPARX-UK)

**REC reference: 22/SW/0149**

**IRAS Project ID: 316644**

This research has been funded by: Medical Research Council (Ref: MR/W002450/1)

Please click YES to each statement below to participate in the study. If there is any statement you don’t agree with, please exit the survey by closing this tab/browser window and you will not take part in the study.

1. I confirm that I have read and understood the information sheet version number [] dated [ ] for the above study. I have had the opportunity to consider the information, ask questions and have had these answered satisfactorily.

**YES**

1. I understand that my participation is voluntary and that I am free to withdraw at any time without giving any reason, without my legal rights being affected. I understand that should I withdraw then the information collected so far cannot be erased and that this information may still be used in the project analysis.

**YES**

1. I understand that relevant sections of my data collected in the study may be looked at by authorised individuals from the University of Nottingham, the research group, and regulatory authorities where it is relevant to my taking part in this study. I give permission for these individuals to have access to these records and to collect, store, analyse and publish information obtained from my participation in this study. I understand that my personal details will be kept confidential.

**YES**

1. I understand that the interview will be audio recorded only and that anonymous direct quotes from the interview may be used in the study reports.

**YES**

5. I agree to take part in the above study.

**YES**

**Please type your name below (this will be taken as your signature to agree to take part in the study)**

Name Date
