# Supplementary material for: Precision computerised cognitive behavioural therapy (cCBT) intervention for adolescents with depression (SPARX-UK): protocol for the process evaluation of a pilot randomised controlled feasibility trial
Source: BMJ Open. 2025 Aug 5;15(8):e092483. doi: 10.1136/bmjopen-2024-092483 (PMC12336577; doi:10.1136/bmjopen-2024-092483)
Supplement: online supplemental file 4 [file bmjopen-15-8-s004.DOCX]

**Good Reporting of A Mixed Methods Study (GRAMMS)**

| **Guideline** | **Section: page** |
| --- | --- |
| Describe the justification for using a mixed methods approach to the research question | Design: p13/14  Strengths and limitations: p14 |
| Describe the design in terms of the purpose, priority and sequence of methods | Design: p13-14 |
| Describe each method in terms of sampling, data collection and analysis | Data collection: p9-11  Data analysis: p11-13 |
| Describe where integration has occurred, how it has occurred and who has participated in it | Design: p14 |
| Describe any limitation of one method associated with the present of the other method | Strengths and limitations: p13-14 |
| Describe any insights gained from mixing or integrating methods | Discussion: p13 and p15-16 |

*O'Cathain A, Murphy E, Nicholl J. The quality of mixed methods studies in health services research. J Health Serv Res Policy. 2008;13(2):92-98.*
