# Supplementary material for: Precision computerised cognitive behavioural therapy (cCBT) intervention for adolescents with depression (SPARX-UK): protocol for the process evaluation of a pilot randomised controlled feasibility trial
Source: BMJ Open. 2025 Aug 5;15(8):e092483. doi: 10.1136/bmjopen-2024-092483 (PMC12336577; doi:10.1136/bmjopen-2024-092483)
Supplement: online supplemental file 5 [file bmjopen-15-8-s005.docx]

**Supplementary File 2**

**ADOLESCENT INTERVIEW SCHEDULE**

**these are topic guides and presented to give the REC an idea of the types of questions/topic areas. The interviews will be semi-structured and thus may not follow this format strictly**

**Preamble**

*Adolescent can have their parent present for the interview if they wish.*

Check that the interviewee has received the information sheet, they/their parent (*depending on age*) has accepted the box stating they are happy to be contacted for an interview on the consent/assent form, understands the SPARX trial and his/her role in it

- Explain that:
  - The aim of the SPARX study was to see the feasibility of conducting a future definitive, larger trial for adolescents with depression.
- The research team is speaking to many people involved in SPARX (e.g., adolescent participants, parents, the e-coach, and clinicians)
- We are interested in your experiences and thoughts about SPARX, so please give honest answers, as both positive and negative feedback will help us improve the intervention and the future trial. You will be asked questions about SPARX.
- However, we will put all the data that we collect in a report to give us an overall picture of SPARX, and no one will be named in the report or know what you answered to the questions, for example, “An adolescent commented that…”

*Ask*: Do you have any initial questions about the study?

**Ethics**

- Remind interviewee:
- The interview will take about 30 minutes
- You do not have to answer any questions that you are not comfortable with and there are no ‘right’ or ‘wrong’ answers
- You can stop at any time, no explanation needed
- If you need a break, please just say, that’s absolutely fine
- If any question doesn’t make sense, ask me to explain

With your permission we are going to record the interview (if telephone then audio only, on a Dictaphone and if Microsoft Teams, video recording) so that we can focus on what you are saying. This will be transcribed by a member of the research team or a company we know well. If you feel more comfortable that we transcribe the interview rather than the company, then we will be happy to do so.

We will delete any mention of places, clinicians/e-coach/family members that may give away yours (or others) name during writing.

The original writing will be put on a password protected hard drive and no one other than members of the research team will be able to view this.

The things you say in the interviews may be used in written reports, published articles and presentations including online but we will never use your name or any other information that may give away who you are.

*Ask*: Do you have any questions about how we use your comments? Please feel free to ask anything however small it may seem at this stage or at any time later.

*Ask*: Is it okay to record the interview?

- If participant not satisfied: answer any questions they have. If they do not want to participate, thank them for their time and finish the interview at this point.

**Explain procedure**

I will begin the interview with my name, the date, time, and the code we have for you - this is just to keep the recordings organised. All your details will be hidden when the interview is written out. The first part will be a little about yourself and your current mood, followed by general questions about the SPARX trial such as what you thought about the study and questionnaires, then moving on to the SPARX intervention and then ending with any recommendations and your overall experience of being involved in SPARX.

*Ask*: Do you have any questions before we start?

*Ask*: Is it okay for me to start recording now?

*State researcher’s name, date, time, and identifying code (for data management)*

**Warm up**

1. Please tell me a little about yourself

- Hobbies/interests?
- Family, things you like to do together.
- School life

1. At the beginning of the study, we sent out information about this study to your mother/father/parents (*personalise according to who the information was sent to*), do you remember if they spoke to you about it and did they speak to you about whether you wanted to participate or not?
2. Can you tell me how your mood currently is?
3. Have you noticed any difference in your mood since you completed SPARX?

- How much better/worse?

First, I am going to ask you questions about being part of the SPARX trial, including how you felt about this and how you found the questionnaires:

**Questions about SPARX as a research project**

1. What did you think about the sound of the SPARX study?

- Who told you about it? (Clinician? Parent?)
- How did you feel about taking part?

1. What did you think of the stuff you had to do before the intervention began?

- Baseline appointment
- Information sheet
- Consent/assent form
- Did you get any help from your parent?

1. Was it clearly explained to you that you would be put in to one of three groups?

- How did you feel about this?
- How did you feel about the group were randomised to?

1. Thinking about what was expected of you during SPARX:

- Can you remember filling in questionnaires? What did you think of them?
- Was the study clearly explained to you?
- How did you feel about completing online questionnaires?
- Has it been okay to manage or a lot of effort? How much help did you need?

Thank you for these answers, that’s been helpful. I’d now like to move on and ask you some questions about how you found the SPARX intervention:

**Questions on SPARX**

1. I am now going to go through the different parts of SPARX and I would like you to tell me how you found them:

- How easy was it to log on? How easy was it to install?
- Did you find it difficult to use anything in SPARX?
- What did you think of the lay out/graphics?
- What did you think of the content/things included?
- Anything that worked particularly well? Anything that could have been improved?
- Did it make sense to you as you did it?
- Any help needed from parents?
- How did you link SPARX into everyday life?

1. Was there anything that stopped you from doing SPARX?

- Busy life? School work?

1. Overall, what did you think about the different sections of SPARX?

- Any sections that you particularly liked or engaged with?

**Prompt**: why?

- Any sections that you did not engage with/found hard?
- Do you think SPARX was too long/too short/just right?

You are now halfway through the questions, so I want you now to think about SPARX and I want to ask you some questions on the impact/effect it has had on you:

1. Before you started SPARX, how did you think it would help your mood and everyday life?

- How good did you expect it to be in improving your mood?
- Did you expect benefits/to help in any other areas of life?

1. With these expectations in mind, how much do you think SPARX has helped you with your mood and in everyday life?
2. How well suited do you feel that SPARX has been in helping you to manage your mood (*Credibility*)?
3. How satisfied were you overall with SPARX (*Satisfaction*)?
4. Which parts of SPARX were particularly helpful to you?
5. Did you have any difficulties with SPARX?

- If so, what were they?
- Technological difficulties? Did you manage to sort them out?
- What did you use to access SPARX? (i.e., tablet/PC/smartphone etc.)

1. Did you adapt/change anything as you went along?
2. Did you apply any of the skills taught in SPARX to your everyday life?

- If so, which ones? Did you find it helpful?
- If not, what stopped you?

1. Did you make any notes as you went along?

- If so, what kind of notes did you make? Relating to specific levels?
- What did you use to make notes? Paper and pen or online etc?

**If adolescent was in the personalised, supported arm: Go to question 21**

**If adolescent was in the unsupported arm: Go to question 23**

1. How did you feel about communicating with (*name of e-coach*)?

- How often did you contact them or how often did they contact you?
- Did you find having the e-coach there helpful/beneficial?
- How did you contact them?
- Which way of contact do you prefer?

1. Did you like having the choices which were presented to you at the beginning?

- Would you have preferred to be given SPARX without any choices?
- Did the personalisation make you more able to engage with SPARX?

Thank you for answering those questions. We only have a few questions left now and these will focus on the future of SPARX:

**Future Direction**

1. How did you feel about getting the intervention online instead of face to face?

**Prompt**: which method do you prefer and why?

1. If we go ahead with our planned larger trial, are there any changes we could make before it is offered to other adolescents?
2. Are there other ways that could be used for giving SPARX to people?

- Any other apps or forms of technology?

1. Overall, how did you feel about the experience of taking part in this study?
2. Would you recommend SPARX to other adolescents with depression?

**Prompt** for clarification of response.

**End of questions**

That reaches the end of the interview and questions I wanted to ask you.

Thank you so much for your time.

- Do you have anything else you wish to speak about that hasn’t been mentioned?
  - Let interviewee talk if they have anything else to add
  - If nothing else – then close interview

If you are okay to end the interview there, I will switch the Dictaphone off/end Teams recording.

*Switch Dictaphone off/press stop recording on Teams*

**Debriefing**

- Ask how they are feeling – whether anything in the interview has troubled them or distressed them or if anything requires clarification
- They or their parents can email me if they have any follow up questions
- Thank them again and ask if they are feeling okay to end interview here.

**PARENT INTERVIEW SCHEDULE**

**these are topic guides and presented to give the REC an idea of the types of questions/topic areas. The interviews will be semi-structured and thus may not follow this format strictly**

**Preamble**

Check that the interviewee has received the information sheet, they have initialled the box stating they are happy to be contacted for an interview on the consent form, understands the SPARX trial and his/her role in it.

- Explain that:
  - The aim of the SPARX study was to see the feasibility of conducting a future definitive, larger trial for adolescents with depression.
- The research team is speaking to many people involved in SPARX (e.g., adolescent participants, parents, the e-coach, and clinicians)
- We are interested in your experiences and thoughts about the SPARX trial, so please give honest answers, as both positive and negative feedback will help us improve the intervention and the future trial.
- However, we will put all the data that we collect in a report to give us an overall picture of SPARX, and no one will be named in the report or know what you answered to the questions, for example, “A parent commented that…”

*Ask*: Do you have any initial questions about the study?

**Ethics**

- Remind interviewee:
- The interview will take about 30 minutes
- You do not have to answer any questions that you are not comfortable with and there are no ‘right’ or ‘wrong’ answers
- You can stop at any time, no explanation needed
- If you need a break, please just say, that’s absolutely fine
- If any question doesn’t make sense, ask me to explain

With your permission we are going to record the interview (if telephone then audio only, on a Dictaphone and if Microsoft Teams, video recording) so that we can focus on what you are saying. This will be transcribed by a member of the research team or a company we know well. If you feel more comfortable that we transcribe the interview rather than the company, then we will be happy to do so.

We will delete any mention of places, clinicians/e-coach/family members that may give away yours (or others) name during writing.

The original writing will be put on a password protected hard drive and no one other than members of the research team will be able to view this.

The things you say in the interviews may be used in written reports, published articles and presentations including online but we will never use your name or any other information that may give away who you are.

*Ask*: Do you have any questions about how we use your comments? Please feel free to ask anything however small it may seem at this stage or at any time later.

*Ask*: Is it okay to record the interview?

- If participant not satisfied: answer any questions they have. If they do not want to participate, thank them for their time and finish the interview at this point.

**Explain procedure**

I will begin the interview with my name, the date, time, and the code we have for your child - this is just to keep the recordings organised. All your details will be hidden when the interview is written out. The first part will be a little about yourself and other studies on depression you may have been involved in, followed by general questions about the study such as how you were recruited and expectations, then moving on to the SPARX intervention more specifically and then ending with any recommendations and your overall experience of being involved in SPARX.

*Ask*: Do you have any questions before we start?

*Ask*: Is it okay for me to start recording now?

*State researcher’s name, date, time, and identifying code (for data management)*

**Warm up**

1. Please tell me a little about yourself and your family

- Family, things you like to do together.

1. Have you and your child ever taken part in any other studies on depression?

- What did that involve?

1. Have you noticed any difference in your child’s mood since they completed SPARX?

- How much better/worse?

First, I am going to ask you questions about being part of the SPARX trial, including how you felt about this and how you found the questionnaires:

**Questions about SPARX as a research project**

1. How did you find out about the SPARX study?

- Who told you about it? (Clinician? Word of mouth?)
- What did you hope to get out of the trial from both you and your child’s point of view?
- What were your initial thoughts about the study?

1. Why did you get involved in this study?
2. What did you think of the way you were approached to take part?

- What did you think of the initial telephone screening?
- Baseline appointment
- Information sheet and consent form
- Thoughts on the whole process
- Anything that you would have liked to be done differently?

1. Was it clearly explained to you that your child would be put in to one of three groups?

- How did you feel about being randomised?
- How did you feel about the group your child was randomised to?

1. Thinking about what was expected of you during SPARX:

- Can you remember filling in questionnaires? What did you think of them?
- Was the study clearly explained to you?
- How did you feel about completing online questionnaires?
- Has it been okay to manage or a lot of effort?
- Did you expect it to take a lot of effort to get your child to engage in questionnaire completion?

Thank you for these answers, that’s been helpful.

**Ask:** *did you view any of the SPARX content?*

**If yes**: Go to question 9

**If no:** Go to question 11

**Questions on SPARX**

1. I am now going to go through the different parts of SPARX and I would like you to tell me how you found them and what you thought your child might have felt about them:

- How easy was it to log on? How easy was it to install? Any difficulties for your child?
- Did your child find it difficult to use anything in SPARX?
- What did you think of the lay out/graphics?
- What did you think of the content/things included?
- Anything that worked particularly well? Anything that could have been improved?
- Did it make sense to your child and you as they did it?
- Did you help your child with any of the content? What aspects needed help?
- How did you and your child link SPARX into everyday life?

1. Overall, what did you think about the different sections of SPARX?

- Any sections that your child particularly liked or engaged with?

**Prompt**: why?

- Any sections that your child did not engage with/found hard?
- Do you think SPARX was too long/too short/just right?

1. Was there anything that stopped your child from doing SPARX?

- Busy life? School work?

1. How did you feel about your level of involvement in SPARX?

- Would you have liked your own content to work through?

You are now halfway through the questions, so I want you now to think about SPARX and I want to ask you some questions on the impact/effect it has had on you and your child:

1. Before you started SPARX, how did you think it would help your child’s mood and everyday life?

- How good did you expect it to be in improving your child’s mood?
- Did you expect benefits/to help in any other areas of your child’s life?

1. With these expectations in mind, how much do you think SPARX has helped your child with their mood and in everyday life?
2. Which parts of SPARX were particularly helpful to your child?
3. Did your child have any difficulties with SPARX?

- If so, what were they?
- Technological difficulties? Did you manage to sort them out?

1. Did your child adapt/change anything as they went along?
2. Would you change anything about SPARX?

- Should we add any information?
- Anything that was not needed?

1. Has being involved in the SPARX trial had any impact on you?

- Has it influenced your approach to your child’s mood?
- Are there any changes/strategies you have made due to SPARX?

**If in the personalised, supported arm go to question 20**

**If in the unsupported arm go to question 22**

1. How did you think your child felt about communicating with (*name of e-coach*)?

- How often did your child contact them or how often did they contact your child?
- Did you think having the e-coach there was helpful/beneficial?
- How did your child contact them?
- Which way of contact do you think they prefer? Which way do you prefer?

1. Do you think your child liked having the choices which were presented at the beginning?

- Do you think they would have preferred to be given SPARX without any choices?
- Do you think personalisation allowed your child to better engage with SPARX?

Thank you for answering those questions. We only have a few questions left now and these will focus on the future of SPARX:

**Future Direction**

1. How did you and your child feel about getting intervention digitally instead of face to face?

**Prompt**: which method do you prefer and why?

1. If we go ahead with our planned larger trial, are there any changes we could make before it is offered to other adolescents?
2. Are there other ways that could be used for giving SPARX to people?

- Any other apps or forms of technology?

1. Overall, how did you feel about the experience of taking part in this study?
2. Would you recommend SPARX to other parents of adolescents with depression?

**Prompt** for clarification of response.

**End of questions**

That reaches the end of the interview and questions I wanted to ask you.

Thank you so much for your time.

- Do you have anything else you wish to speak about that hasn’t been mentioned?
  - Let interviewee talk if they have anything else to add
  - If nothing else – then close interview

If you are okay to end the interview there, I will switch the Dictaphone off/end Teams recording.

*Switch Dictaphone off/press stop recording on Teams*

**Debriefing**

- Ask how they are feeling – whether anything in the interview has troubled them or distressed them or if anything requires clarification
- They can email me if they have any follow up questions
- Thank them again and ask if they are feeling okay to end interview here.

**E-COACH INTERVIEW SCHEDULE**

**these are topic guides and presented to give the REC an idea of the types of questions/topic areas. The interviews will be semi-structured and thus may not follow this format strictly**

**Preamble**

- Explain that:
- We are interested in individual experiences and thoughts about SPARX, so please give honest responses, as both positive and negative feedback will help us improve the intervention and trial. Explain that they will be asked questions relating to their involvement in SPARX, their thoughts on the intervention, feedback they received, experience of supervision, and any recommendations they may have
- However, we combine all the data we collect to provide an overall picture of SPARX and its implementation. Any comments in the report are attributed very generally, for example, “The e-coach commented that…” All comments/opinions will be strictly confidential.

*Ask*: Do you have any initial questions?

**Ethics**

- Remind interviewee:
- The interview will take about 25 minutes
- You do not have to answer any questions that you are not comfortable with and there are no ‘right’ or ‘wrong’ answers
- You can stop at any time, no explanation needed
- If you need a comfort/loo break, please just say, that’s absolutely fine
- If any question doesn’t make sense, ask for an explanation

With your permission we are going to record the interview (if by telephone, audio only, on a Dictaphone and if by Microsoft Teams, video recording) so that we can focus on what you are saying. This will be transcribed by a member of the research team or an approved company.

We remove any reference to any places, clinicians/therapists/family members that may give away yours (or others) identity during transcription.

The original transcription will be stored on an encrypted hard drive and no one other than immediate members of the research team can access this.

*Ask*: Do you have any questions about how we use your comments?

*Ask*: Is it okay to record the interview?

- If participant not satisfied: answer any questions they have. If they do not want to participate, thank them for their time and finish the interview at this point.

**Explain procedure**

I will begin the interview with my name, the date, and time - this is just to keep the recordings organised. All your details will be anonymised when the data is transcribed. The first part will be a little about yourself and your role in SPARX, followed by general questions about the SPARX trial, then moving on to the SPARX intervention more specifically and then ending with any recommendations and your overall experience of being involved in SPARX.

*Ask*: Do you have any questions before we start?

*Ask*: Is it okay for me to start recording now?

*State researcher’s name, date, and time (for data management)*

I want to start by asking a bit about you:

**Background Questions**

1. Please briefly describe your professional background

- Profession
- If applicable, how long have you worked with adolescents who have mental health difficulties?

1. What previous experience did you have that was relevant to your role as an e-coach on SPARX?

**Prompt**

- Training
- Education

1. What were your thoughts about computerised interventions before you began in this role?

Now I am going to ask you about your involvement in the trial:

**Questions about SPARX as a trial**

1. How did you find out about the SPARX trial?
2. Why did you get involved in this trial?

- How did you feel about being involved?

1. Are there any specific challenges for the e-coach because the intervention is being delivered as part of a trial?

**Prompt**

- Keeping interventions separate/avoiding contamination
- Rigour of protocol
- Having sufficient time to adhere to the protocol
- Sense that you are offering a helpful, effective intervention

Thank you for these answers, that’s been really helpful. I’d now like to move on and ask you some questions on the role of the e-coach and its subsequent demands:

**E-coach role and demands**

1. Can you tell me a bit about the role of the e-coach in assisted computerised interventions?

**Prompt**

- Any advantages/rewarding aspects to the role (e.g., convenience, job satisfaction etc.)?
- Any limitations (e.g., safety issues, feasibility etc.)?
- Any suggestions for overcoming identified limitations?

1. What personal skills/experience do you think are needed for the e-coach to effectively support SPARX?

- How experienced do you think they need to be?
- Experience with digital interventions?
- Knowledge of CBT?
- Experience working with adolescents?
- Are there any specific challenges because the intervention is with adolescents?
- Prior clinical training?

1. Are there any training needs you can identify that may aid the e-coach in supporting SPARX?

**Prompt**

- Knowledge of depression and mental health conditions
- Training in computerised interventions
- Training in CBT

1. How have you structured/managed this role alongside your other commitments?

- How many hours did you dedicate to SPARX per day?
- How has the workload felt?
- Has anything felt particularly difficult/stressful?
- Were there any tools you used/found useful that helped manage your workload? (e.g., excel sheets)
- Are there any structural changes you can identify that would make the e-coaches role more effective/manageable?

1. Can you please share your experiences of the supervision you received?

**Prompt**

- Quality of sessions – what went well/less well?
- Quantity of sessions
- Structure – how did the supervision work?
- Common issues that arose – general trial issues vs delivery of the intervention issues?

Now I would like to ask you some questions about the delivery of SPARX:

**Perceptions of SPARX delivery**

1. Do you believe that SPARX is being delivered as planned?

- If so, how? If not, why?
- Predictable outcomes?
- Any unanticipated consequences?

1. How do you see the role of the e-coach in the supported, personalised arm?

**Prompt**

- What sort of support do the adolescents need?
- Anything you felt went particularly well?
- Any difficulties?

1. How do you feel about supporting adolescents in computerised interventions?

- What are the benefits/limitations?

1. What feedback have you received from adolescents/parents?

**Prompt**

- Benefits
- Problems encountered
- Frustrations
- Participant characteristics that influenced their feedback (e.g., age/gender?)

1. How often did you interact with participants?

**Prompt**

- Daily/every other day/once a week/twice a week?
- What were the main types of comments you received?
- Was it manageable?
- What format were these interactions (e.g., phone, messages)?
- Were there any difficulties in responding to participants?

1. How do you feel about the relationships you have developed with adolescents and their parents?

- Did the interactions feel meaningful?
- Was this more difficult to develop online than F2F?
- Did this hinder/help the effectiveness and implementation of SPARX?

1. What is your view SPARX, and the things included in it?

**Prompt**

- Structure
- Content
- Language/flow
- Too long/too short?

1. Would you change anything about SPARX?

- Additional information? Unnecessary information?

1. Why do you think adolescents may not wish to engage/persist with SPARX?

**Prompt**

- Barriers?
- Could we have done anything differently?
- How can we better engage them in future work?
- Characteristics of those who found it difficult to engage (e.g., age/gender)?

1. What do you believe were the main barriers to effectively implementing SPARX?

- Internal/external factors?
- Any solutions?

1. What do you think have been the overall effects of SPARX on participants?
2. What did you think of the personalisation aspect of SPARX?

- Any benefits?

1. Do you think face to face interventions are more effective than digital?

- If so, why?
- A combination of the two?
- Do you think a face-to-face intervention may have given us a different outcome?

Thank you for answering those questions. We only have a few questions left now and these will focus on the future of SPARX and your overall thoughts:

**Future Direction**

1. Is there anything else we could have done differently?
2. Do you think if we are to go ahead as planned with our larger trial, SPARX should be purely supported and personalised or purely self-directed?

- Why?

1. Overall, would you recommend SPARX to adolescents with depression?

- Why?
- At what point of diagnosis/age?

**End of questions**

That reaches the end of the interview and questions I wanted to ask you.

Thank you very much for your time.

- Do you have anything else you wish to speak about that hasn’t been mentioned?
  - Let interviewee talk if they have anything else to add
  - If nothing else – then close interview

If you are okay to end the interview there, I will switch the Dictaphone off/end Teams recording.

*Switch Dictaphone off/Stop recording on Teams*

**Debriefing**

- Ask how they are feeling and if anything requires clarification
- They can email me if they have any follow up questions/comments

Thank them again, and ask if they are feeling okay to end

**CLINICIAN INTERVIEW SCHEDULE**

**these are topic guides and presented to give the REC an idea of the types of questions/topic areas. The interviews will be semi-structured and thus may not follow this format strictly**

**Preamble**

- Check that the interviewee has received the information sheet, understands the SPARX trial and his/her role in it
- Explain that:
  - The aim of the SPARX trial was to investigate whether a computerised CBT serious game could be progressed to a full definitive larger randomised controlled trial.
- The research team is speaking to a range of people involved in SPARX (e.g., adolescents and parents, the e-coach, and clinicians)
- We are interested in individual experiences and thoughts about SPARX, so please give honest responses, as both positive and negative feedback will help us improve the intervention and trial going forward. Explain that they will be asked questions relating to their involvement in the SPARX trial, experiences with recruitment, and factors relating to their institution (e.g., NHS/MHST)
- However, we combine all the data we collect to provide an overall picture of SPARX and its implementation and any comments in the report are attributed very generally, for example, “A clinician commented that…” All comments/opinions will be strictly confidential.

*Ask*: Do you have any initial questions about the project?

**Ethics**

- Remind interviewee:
- The interview will take about 20 minutes
- You do not have to answer any questions that you are not comfortable with and there are no ‘right’ or ‘wrong’ answers
- You can stop at any time, no explanation needed
- If you need a comfort/loo break, please just say, that’s absolutely fine
- If any question doesn’t make sense, ask for an explanation

With your permission we are going to record the interview (if by telephone, audio only, on a Dictaphone and if by Microsoft Teams, video recording) so that we can focus on what you are saying. This will be transcribed by a member of the research team or an approved company.

We remove any reference to any places, therapists/family members that may give away yours (or others) identity during transcription.

The original transcription will be stored on an encrypted hard drive and no one other than immediate members of the research team can access this.

*Ask*: Do you have any questions about how we use your comments?

*Ask*: Is it okay to record the interview?

- If participant not satisfied: answer any questions they have. If they do not want to participate, thank them for their time and finish the interview at this point.

**Explain procedure**

I will begin the interview with my name, the date, and time - this is just to keep the recordings organised. All your details will be anonymised when the data is transcribed. The first part will be a little about yourself, followed by general questions about the SPARX trial, moving on to your views on recruitment, and ending with institutional issues and future direction.

*Ask*: Do you have any questions before we start?

*Ask*: Is it okay for me to start recording now?

*State researcher’s name, date, and time (for data management)*

I want to start by asking some questions about you:

**Background Questions**

1. Please briefly describe your professional background

**Prompt** (if not covered)

- What is your job title?
- How long have you worked as a clinician/practitioner with children/adolescents?
- How much contact do you normally have with adolescents with depression?

1. What treatment recommendations would you normally prescribe for adolescents with depression?

**Prompt** (if not covered)

- Referral to face-to-face therapist?

1. How long is the waiting list for face-to-face CBT for adolescents with depression?

Now I am going to ask you questions about being part of the SPARX trial:

**Questions about SPARX as a trial**

1. How did you find out about the SPARX trial?
2. Why did you get involved in this trial?
3. How did you feel about being involved in this trial?
4. What were your expectations of the SPARX trial?

- Did it sound like something that would be effective?
- Did you expect people to take part?

Thank you for these answers, that’s been really helpful. I’d now like to move on and ask about your thoughts on recruitment for the trial:

**Recruitment for SPARX**

1. What was your experience of recruiting participants to the SPARX trial?

**Prompt**

- Did it take a lot of time?
- How difficult was it to recruit to the trial?
- Were all the procedures (e.g., returning of consent to contact forms) clear?

1. Were there any factors that affected recruitment?
   **Prompt**

- Drivers to recruitment?
- Ability to offer a service for patients?
- Barriers?
- Employ any strategies to improve recruitment?

1. What factors influenced whether you approached a family about the trial?

**Prompt**

- Having the materials to hand?
- Remembering at the time?
- Characteristics of the family that you approached (e.g., engaged parents or those not currently in crises)?
- Availability of treatment options in your own clinic and locally

1. Why do you think some adolescents/parents may not have agreed to take part?
2. Why do you think adolescents/parents may not have persisted with SPARX?

- How can we better engage adolescents and families in future work?

1. Have you received any feedback from parents/adolescents about the SPARX trial?

- If so, what was it?

1. Do you think face to face therapy is more effective than digital?

- If so, why?

1. Overall, how do you feel about inviting patients to participate in studies external from your care?

**Prompt**

- Motivations for doing so?
- Is the fact the therapy is delivered externally a positive? Why?

Thank you for answering those questions. We only have a few questions left now and these will focus on institutional factors:

**Institutional factors**

1. What, if anything, have you/your team/your clinic learnt from being involved in this trial?

- Anyone else from team involved?

1. How do you think the NHS/MHST could incorporate the SPARX intervention into everyday practice?

**Prompt**

- Feasibility
- Benefits
- Obstacles

1. Do you think the NHS would be able to/willing to fund such a project?

- Costs versus benefits
- Good use of money?

**Future Direction**

1. Is there anything we could have done differently on this trial?
2. Overall, would you recommend SPARX to adolescents with depression?

- Why?
- At what point of diagnosis/age?

**End of questions**

That reaches the end of the interview and questions I wanted to ask you.

Thank you very much for your time.

- Do you have anything else you wish to speak about that hasn’t been mentioned?
  - Let interviewee talk if they have anything else to add
  - If nothing else – then close interview

If you are okay to end the interview there, I will switch the Dictaphone off/stop recording.

*Switch Dictaphone off/stop Teams recording*

**Debriefing**

- They can email me if they have any follow up questions/comments
- Thank them again and ask if they are feeling okay to end interview here.
