# Supplementary material for: Precision computerised cognitive behavioural therapy (cCBT) intervention for adolescents with depression (SPARX-UK): protocol for the process evaluation of a pilot randomised controlled feasibility trial
Source: BMJ Open. 2025 Aug 5;15(8):e092483. doi: 10.1136/bmjopen-2024-092483 (PMC12336577; doi:10.1136/bmjopen-2024-092483)
Supplement: Supplementary file 6 [file bmjopen-2024-092483.reviewer_comments.pdf]

PEER REVIEW HISTORY

BMJ Open publishes all reviews undertaken for accepted manuscripts. Reviewers are asked to complete a checklist review form and are provided with free text boxes to elaborate on their assessment. These free text comments are reproduced below.

ARTICLE DETAILS

Title (Provisional)

A Precision Computerised Cognitive Behavioural Therapy (cCBT) Intervention for Adolescents with Depression (SPARX-UK): Protocol for the Process Evaluation of a Pilot Randomised Controlled Feasibility Trial

Authors

Khan, Kareem; Babbage, Camilla May; Sprange, Kirsty; Hall, Charlotte Lucy; Parker, Adam; Greenhalgh, Chris; Jeffery, Matthew; Lucassen, Mathijs; Merry, S; Prasad, V; Stasiak, Karolina; Guo, Boliang; Tench, Christopher R.; Wright, Hannah; Stallard, Paul; Hollis, Chris; Youth, Digital

---

VERSION 1 - REVIEW

**Reviewer**	**1**
**Name**	**Richards, David**
**Affiliation**	**University of Exeter, Institute for Health Research**
**Date**	**06-Jan-2025**
**COI**	**None**

The following suggestions are intended to improve your paper, which currently has a considerable number of issues that require your attention.

1. Ordinarily we write such papers in the first person (in this case first person future tense). I suggest you edit the text throughout to this effect.

2. Referencing is problematic. a) on p3 you cite reference 18 as evidence that the intervention has an effect. In fact this reference is a protocol and so unable to fulfil this objective. Next you cite a review that claims your intervention is effective but this was published before the previous reference 18 (which you have erroneously cited as evidence of effect anyway). In fact, you only cite one paper on the intervention from 2012, a severely underpowered study of less than 100 participants per arm for a non-inferiority trial planned for 600 participants originally. One wonders where the statement 'efficacy in various studies' comes from. Please provide more robust citations and give the effect sizes from these evaluations in your background text. b) you do not cite the latest (now quite old) 2021 Skivington et al version of the MRC guidelines. Please remedy this and consider the advice

within it on designing complex intervention evaluations, including process evaluations. Moore et al is now ten years old and has been developed further.

3. Research questions are unclear. Beneath a general aim, it would be helpful if you could articulate clear research questions which are designed to specifically answer key process evaluation questions. These should be directed at a) fidelity, b) mechanisms, c) context. They should also be directly related to factors identified in your logic model. Currently there is no red thread connecting these elements of your paper, including into the measurement protocol later.

4. Please justify your sample size for the trial and your sample sizes for the various data collections in your process evaluation.

5. Your logic model is non-standard and confusing. a) Your impact column refers to the impact of your trial, including data from your intended process evaluation, not the impact of your intervention (greater population wellbeing, reduced prevalence of depression for example....). b) you cite 'mediators and moderators' in your outputs column, which makes no sense to me. What are they? What do you hypothesise will drive outputs? To assist you, please note that CBT has very specific hypothesised behavioural and cognitive mechanisms. Supported CBT has very specific relational mechanism hypotheses and in your case also mechanisms around the gamification of the intervention which is explicitly designed to increase engagement and intervention retention. Your logic model currently makes no mention of these factors, which is a severe omission. They should drive process evaluation data collection (see later point). c) you mention 'moderators' in the outputs column as noted above (which requires correction), but individual moderators are not included in your context box. These could include multiple factors known to drive outcomes in CBT - sex/gender, age, baseline depression severity, symptom profiles, previous episodes, concurrent pharmacological treatment, etc. Amend please. And then later include these selected variables in your measurement and analyses sections.

6. As written, this protocol reads almost exclusively as a qualitative acceptability study. The data collection proposals and measures you cite do not follow the red threads I suggested earlier from questions driven by a logic model to address fidelity, mechanisms and contextual influences on outcomes. I suggest you have a choice: either rewrite the protocol as a qualitative acceptability study, or add variables and analyses organised around classic process evaluation structures. To do this you need to identify mechanistic measures and individual context measures and a clearer account of how you will measure fidelity. Then, most importantly of all, we need to see how you will use this data to make predictions about how these various factors drive outcomes. You have written (p14) that you will examine contextual variables' using linear regression but as currently written we do not know what these variables are, nor how you will regress them against what outcomes. The whole point of a process evaluation, even a qualitative one, is to identify influences on outcomes. This is not the case in your current manuscript.

7. A more minor comment, which you may not feel is of interest should you choose to amend as suggested above, is that it seems somewhat strange that you want to 'survey' drop outs but interview the rest. Why not interview both groups, given you have ethics for drop outs? Indeed, what is your criteria for drop out of trial compared to criteria for drop out of the intervention, both of which are very different concepts?

8. With any study collecting both narrative and numerical data, mixing them is desirable but an underdeveloped methodological science. Triangulation is not actually a relevant concept. If you want to mix analysis which model (e.g. the many suggested by Plano Clark etc.) will you use? How will you link data? By individual or by group? We need far more detail on your planned methods here.

9. A final minor issue is your use of the word 'causes' on p8 in relation to behaviour change. Even in a process evaluation of a fully powered trial, the process evaluation will never be powered to determine causes. It can only suggest potential relationships between factors on an observational level.

In summary, as a process evaluation protocol your manuscript can be greatly improved if you identify clear research questions on fidelity, mechanisms and context related closely to an amended logic model, which you should follow through with specific measurements and analyses. Or rewrite as a qualitative acceptability study, scaling back your ambition to just this one aspect of a process evaluation.

**Reviewer**	**2**
**Name**	**Andersson, Rebecca**
**Affiliation**	**Karolinska Institutet, Department of Clinical Neuroscience**
**Date**	**26-Feb-2025**
**COI**	**None**

Dear authors,

This is a very interesting protocol for a planned study, which has the capacity to contribute to the field of digital interventions by enhancing our understanding of why a certain intervention works or not. You should be commended for your work on patient and public involvement, as well as attempting to understand the reasons behind not only treatment success but also drop-outs from treatment, and for providing a critical view of the risk of producing skewed results and for the measures taken to balance this.

When I refer to pages in my comments below, I am talking about the pages in the upper right or left corner.

Kind regards,

Rebecca Andersson

Clinical psychologist and doctoral student

Karolinska Institutet

COMMENTS:

Abstract

- Please clarify whether you are comparing guided versus self-directed or guided versus control and self-directed versus control. I think comparing the experimental group/s with the control group rather than contrasting the two experimental groups against one another is more conventional.

- Consider mentioning thematic analysis (not only “analysed thematically”) when describing your methods in the abstract. I first encounter it in the Methods section, alongside the framework analysis method (the latter being a subtype of thematic analysis?).

Introduction

- Include a definition of serious games in the introduction. Now, you explain the concept behind serious games and describe SPARX as an example of serious games, but I would appreciate a definition added as well.

- In this study, you plan to evaluate supported and unsupported/self-directed cCBT. I believe this can be emphasised in the introduction, for instance by answering: What is currently known about the impact of support on cCBT, and what is the effect of unsupported cCBT for youth? How will the different versions be assessed in the process evaluation?

- P 6, rows 56-57: What is meant by “no additional therapeutic input”? Does this imply no techniques beyond the online material? This could be clarified slightly. How will treatment adherence be evaluated? Perhaps this is stated in your previous study protocol and is beyond the scope of this protocol that describes the process evaluation?

- Is this treatment a preventive intervention or a treatment intervention? Considering the criteria for the study (symptoms of depression rather than diagnosed depression), it seems to me to be a preventive intervention. If so, please change throughout the paper.

- Please add that adolescents under 16 need to assent and that it is, I assume, required for both the parent/guardian and the adolescent to consent to the study for participation to be possible. In my view, it is important to obtain assent/consent from both parties to respect the adolescent’s autonomy and to avoid involuntary participation on their part.

- P 7, lines 49-53: How did you arrive at the sample size? My impression is that it appears somewhat high for a feasibility study but too low to effectively address efficacy questions (which, as I understand it, is not your aim). Please provide a justification for your chosen sample size.

- P 8, lines 3-5: This is a very interesting aim in your study: “This will be achieved by

seeking to understand the causes of the behaviour change data obtained from the trial." I think it would be even more interesting if you also tried to understand the reasons for the lack of behaviour change (since we know that a significant portion of patients do not improve after depression treatment).

Methods

- Again, this is perhaps beyond the scope of this protocol, so please excuse me if so. But I would like to see a rationale for choosing the waitlist as your control group. It is sometimes questionable to use a control group that is less effective than existing treatments, particularly from an ethical standpoint. Please refer to Gold et al. (2017), which state that a comparison group needs to be safe, potentially effective, and desirable.

Gold, S. M., Enck, P., Hasselmann, H., Friede, T., Hegerl, U., Mohr, D. C., & Otte, C. (2017). Control conditions for randomised trials of behavioural interventions in psychiatry: a decision framework. The Lancet Psychiatry, 4(9), 725-732.

- P 10, lines 40-44: add a reference already in the first sentence where thematic analysis is mentioned: "As the process evaluation is a combination of exploration and description, thematic analysis will be used to identify, analyse, and report patterns within the transcribed interviews (REF)."

- P 12, lines 40-41. I don't disagree with you, but could you please elaborate on what you mean when you say that thematic analysis is regarded as the most flexible qualitative analytical process?

- You plan independent chi-squared tests and between-group t-tests. However, let's consider if you have enough power to compare groups accurately. This usually requires larger sizes per group when evaluating depression treatment (due to spontaneous remission and moderate effectiveness in general). A rough estimate is that around 75 to 100 participants per each group. It might be a good idea to stick with within-group t-tests for now and keep those between-group comparisons in mind for a future, larger-scale trial. I'd love to hear your thoughts if you see things differently.

- P 14, lines 7-8. Please specify the contextual variables you will examine using multiple linear regression models. A brief mention in parentheses would suffice.

- P 15, lines 9-10. Kindly include your plan for disseminating the results of this study.

- Methods: I would like to see some details on participant recruitment for the trial. Please include this or direct me to where I can find it. Under "Sampling and recruitment for adolescent and parent/guardian interviews," I found recruitment plans for the qualitative interviews but not for the trial itself. Perhaps this again because this information belongs to the trial protocol?

- I also miss a description of how you plan to manage safety in this study (how to assess and monitor suicidality, how to monitor and report adverse events, etc.). Perhaps this was left

out intentionally since it is a protocol for the process evaluation. If so, could you refer to your full study protocol and add this to the supplements, maybe? study protocol (18) (26/03/2024).

Minor comment

- Page 5, line 5-6 : Should it be characterised rather than categorised? I believe also add “a” before persistent mood.

VERSION 1 - AUTHOR RESPONSE

Reviewer: 1

Comments to the Author:

The following suggestions are intended to improve your paper, which currently has a considerable number of issues that require your attention.

1. Ordinarily we write such papers in the first person (in this case first person future tense). I suggest you edit the text throughout to this effect.

- *We thank the reviewer for this suggestion and have edited the manuscript throughout to reflect this suggestion in terms of our writing style.*

2. Referencing is problematic. a) on p3 you cite reference 18 as evidence that the intervention has an effect. In fact this reference is a protocol and so unable to fulfil this objective. Next you cite a review that claims your intervention is effective but this was published before the previous reference 18 (which you have erroneously cited as evidence of effect anyway). In fact, you only cite one paper on the intervention from 2012, a severely underpowered study of less than 100 participants per arm for a non-inferiority trial planned for 600 participants originally. One wonders where the statement 'efficacy in various studies' comes from. Please provide more robust citations and give the effect sizes from these evaluations in your background text.

- *We thank the reviewer for pointing this out and have now added more robust citations with effect sizes which contextualise the efficacy of SPARX on page 5. However, we have mentioned that most studies which showed efficacy were conducted in Australasia and studies outside of that region have failed to show efficacy. Hence, the need for an evaluation in the UK.*

b) you do not cite the latest (now quite old) 2021 Skivington et al version of the MRC guidelines. Please remedy this and consider the advice within it on designing complex intervention evaluations, including process evaluations. Moore et al is now ten years old and has been developed further.

- *We have now added the Skivington et al. (2021) reference on page 6, however, our process evaluation follows the guidelines from Moore et al. (2015) as these are still considered to be the definitive guidelines for conducting process evaluations.*

3. Research questions are unclear. Beneath a general aim, it would be helpful if you could articulate clear research questions which are designed to specifically answer key process evaluation questions. These should be directed at a) fidelity, b) mechanisms, c) context. They should also be directly related to factors identified in your logic model. Currently there is no red thread connecting these elements of your paper, including into the measurement protocol later.

- *We thank the reviewer for this helpful suggestion and have now added our specific research questions based on the three process evaluation components on pages 8-9.*

4. Please justify your sample size for the trial and your sample sizes for the various data collections in your process evaluation.

- *We have now justified the sample size for the main trial on page 7 and have justified the sample sizes for the qualitative sample on pages 14 and 15.*

5. Your logic model is non-standard and confusing. a) Your impact column refers to the impact of your trial, including data from your intended process evaluation, not the impact of your intervention (greater population wellbeing, reduced prevalence of depression for example....). b) you cite 'mediators and moderators' in your outputs column, which makes no sense to me. What are they? What do you hypothesise will drive outputs? To assist you, please note that CBT has very specific hypothesised behavioural and cognitive mechanisms. Supported CBT has very specific relational mechanism hypotheses and in your case also mechanisms around the gamification of the intervention which is explicitly designed to increase engagement and intervention retention. Your logic model currently makes no mention of these factors, which is a severe omission. They should drive process evaluation data collection (see later point). c) you mention 'moderators' in the outputs column as noted above (which requires correction), but individual moderators are not included in your context box. These could include multiple factors known to drive outcomes in CBT - sex/gender, age, baseline depression severity, symptom profiles, previous episodes, concurrent pharmacological treatment, etc. Amend please. And then later include these selected variables in your measurement and analyses sections.

- *We thank the reviewer for these helpful suggestions and agree the logic model needed improving. We have now updated the logic model including edits made to the context (relating to participant characteristics, social and environmental factors and structural considerations), inputs (including intervention, implementation and evaluation frameworks), outputs (now including intervention engagement, therapeutic processes, moderators and mediator headings) and impact boxes (relating to primary and secondary outcomes, and long-term impact). We have also added a new mechanism of impact box highlighting the CBT, relational and gamification mechanisms of impact (see Figure 1, page 10). We hope these additions have improved its coherence.*

6. As written, this protocol reads almost exclusively as a qualitative acceptability study. The data collection proposals and measures you cite do not follow the red threads I suggested earlier from questions driven by a logic model to address fidelity, mechanisms and contextual influences on outcomes. I suggest you have a choice: either rewrite the protocol as a qualitative acceptability study, or add variables and analyses organised around classic process evaluation structures. To do this you need to identify mechanistic measures and individual context measures and a clearer account of how you will measure fidelity. Then, most importantly of all, we need to see how you will use this data to make predictions about how these various factors drive outcomes. You have written (p14) that you will examine contextual variables' using linear regression but as currently written we do not know what these variables are, nor how you will regress them against what outcomes. The whole point of a process evaluation, even a qualitative one, is to identify influences on outcomes. This is not the case in your current manuscript.

- *We thank the reviewer for this point and hope that the manuscript has been significantly improved by following the other recommendations throughout. We have now added research questions, updated the logic model, and added which contextual variables we will analyse in the linear regression.*

7. A more minor comment, which you may not feel is of interest should you choose to amend as suggested above, is that it seems somewhat strange that you want to 'survey' drop outs but interview the rest. Why not interview both groups, given you have ethics for drop outs? Indeed, what is your criteria for drop out of trial compared to criteria for drop out of the intervention, both of which are very different concepts?

- *The reason why we are aiming to survey drop outs is that from our experience of conducting previous process evaluations participants who drop out do not agree to be interviewed. Thus, by administering a short survey which is sent automatically to those who drop out (of both those who withdraw from the intervention alone and those who withdraw from the trial) we are able to capture the views of those who drop out as well as those who remain involved in the trial, so as not to bias the evaluation towards more positive experiences. Our first option is to interview dropouts, however, by having this survey available this gives us another option to capture their experiences.*

8. With any study collecting both narrative and numerical data, mixing them is desirable but an underdeveloped methodological science. Triangulation is not actually a relevant concept. If you want to mix analysis which model (e.g. the many suggested by Plano Clark etc.) will you use? How will you link data? By individual or by group? We need far more detail on your planned methods here.

- *We appreciate the reviewer's insightful feedback regarding the integration of qualitative and quantitative data. We have now clarified our mixed-methods approach by specifying the framework we will use for data integration. Instead of relying on triangulation, we will adopt a convergent mixed-methods design (Creswell & Plano Clark, 2018), where qualitative and quantitative data are analysed separately and then merged for interpretation.*
- *To link the data, we will use a group-level integration strategy, meaning that we will match qualitative interview responses with the corresponding quantitative trial data for the same groups (i.e. supported versus unsupported). This allows us to explore and compare how fidelity, mechanisms, and contextual influences relate to observed trial outcomes in the two groups. We will present these findings using joint displays, a recommended technique for visualising integrated mixed-methods results (Guetterman et al., 2015).*

References:

Creswell, J. W., & Plano Clark, V. L. (2017). Designing and Conducting Mixed Methods Research (3rd ed.). SAGE Publications.

Guetterman, T. C., Fetters, M. D., & Creswell, J. W. (2015). Integrating Quantitative and Qualitative Results in Health Science Mixed Methods Research Through Joint Displays. Annals of Family Medicine, 13(6), 554–561. https://doi.org/10.1370/afm.1865

9. A final minor issue is your use of the word 'causes' on p8 in relation to behaviour change. Even in a process evaluation of a fully powered trial, the process evaluation will never be powered to determine causes. It can only suggest potential relationships between factors on an observational level.

- *We thank the reviewer for pointing this out and we have now edited this sentence to read, "This will be achieved by seeking to understand potential relationships between factors obtained from trial data" on page 8*

In summary, as a process evaluation protocol your manuscript can be greatly improved if you identify clear research questions on fidelity, mechanisms and context related closely to an amended logic model, which you should follow through with specific measurements and analyses. Or rewrite

as a qualitative acceptability study, scaling back your ambition to just this one aspect of a process evaluation.

- *We thank the reviewer for their helpful suggestions which we feel have improved the quality of our manuscript and the overall conduct of the process evaluation.*

Reviewer: 2

Comments to the Author:

Dear authors,

This is a very interesting protocol for a planned study, which has the capacity to contribute to the field of digital interventions by enhancing our understanding of why a certain intervention works or not. You should be commended for your work on patient and public involvement, as well as attempting to understand the reasons behind not only treatment success but also drop-outs from treatment, and for providing a critical view of the risk of producing skewed results and for the measures taken to balance this.

- *We thank the reviewer for their kind and encouraging comments about our manuscript.*

Abstract

- Please clarify whether you are comparing guided versus self-directed or guided versus control and self-directed versus control. I think comparing the experimental group/s with the control group rather than contrasting the two experimental groups against one another is more conventional.

  - *We have stated in the Abstract that, in the main trial, we are comparing supported versus self-directed versions of SPARX, and have now clarified that we are doing this so we can see which version we should proceed with in the definitive RCT (if such a trial is indicated, based on the results of the pilot study).*

- Consider mentioning thematic analysis (not only “analysed thematically”) when describing your methods in the abstract. I first encounter it in the Methods section, alongside the framework analysis method (the latter being a subtype of thematic analysis?).

  - *We thank the reviewer for this point and have now added we will be analysing the data using thematic analysis using a framework approach on page 2.*

Introduction

- Include a definition of serious games in the introduction. Now, you explain the concept behind serious games and describe SPARX as an example of serious games, but I would appreciate a definition added as well.

  - *We have now added such a definition which is “games that do not have entertainment, enjoyment, or fun as their primary purpose (Michael and Chen, 2005)” on page 5.*

Reference:

D. R. Michael and S. L. Chen, Serious Games: Games That Educate, Train, and Inform, Muska & Lipman/Premier-Trade, 2005.

- In this study, you plan to evaluate supported and unsupported/self-directed cCBT. I believe this can be emphasised in the introduction, for instance by answering: What is currently known about the impact of support on cCBT, and what is the effect of unsupported cCBT for youth? How will the different versions be assessed in the process evaluation?

- *We thank the reviewer for this important point and have now added the following to the Introduction on page 4: "Despite the efficacy of DHIs such as cCBT, there have been several issues with low engagement and adherence with unsupported interventions. For example, a study evaluating a self-directed DHI called MoodGYM in secondary schools found that only 8.5% of participants logged on to use the intervention (Lillevoll et al., 2014). Indeed, it is widely known that a commonly purported facilitator that improves engagement and adherence with DHIs is human support (Baumeister et al., 2014)."*
- *In line with reviewer 1's comment, we have now clarified that we will use the mixed methods process evaluation data to compare the two versions of SPARX (see page 17).*

References:

Lillevoll KR, Vangberg HCB, Griffiths KM, Waterloo K, Eisemann MR. Uptake and adherence of a self-directed Internet-based mental health intervention with tailored e-mail reminders in senior high schools in Norway. BMC Psychiatry. 2014;14:14. Available from: http://www.ncbi.nlm.nih.gov/pubmed/24443820

Baumeister, H., Reichler, L., Munzinger, M., and Lin, J. (2014). The impact of guidance on Internet-based mental health interventions—a systematic review. Internet Interventions, 1, 205–215. doi: 10.1016/j.invent.2014.08.003

- P 6, rows 56-57: What is meant by "no additional therapeutic input"? Does this imply no techniques beyond the online material? This could be clarified slightly. How will treatment adherence be evaluated? Perhaps this is stated in your previous study protocol and is beyond the scope of this protocol that describes the process evaluation?

- *We have now clarified that "no additional therapeutic input" means that no therapeutic techniques will be specified beyond the online material provided as part of SPARX on page 7.*
- *You are correct that treatment adherence has been stated in our published study protocol and has been defined as the percentage completing four levels or more of SPARX*.

- Is this treatment a preventive intervention or a treatment intervention? Considering the criteria for the study (symptoms of depression rather than diagnosed depression), it seems to me to be a preventive intervention. If so, please change throughout the paper.

- *We thank the reviewer for raising this interesting point. The intervention targets those with symptoms of depression and we are primarily recruiting from clinical services, so we anticipate that the intervention will be treatment for these young people. On the other hand, we are also recruiting young people from outside of clinical routes or young people on waiting lists. These young people are unlikely to have a confirmed diagnosis, though will have elevated symptoms of depression. Hence, we have not referred to this as either a treatment or a preventive intervention.*

- Please add that adolescents under 16 need to assent and that it is, I assume, required for both the parent/guardian and the adolescent to consent to the study for participation to be possible. In my view, it is important to obtain assent/consent from both parties to respect the adolescent's autonomy and to avoid involuntary participation on their part.

- *We thank the reviewer for pointing this out and have now clarified that adolescents under 16 need to provide assent (see page 7).*

- P 7, lines 49-53: How did you arrive at the sample size? My impression is that it appears somewhat high for a feasibility study but too low to effectively address efficacy questions (which, as I understand it, is not your aim). Please provide a justification for your chosen sample size.

- *In line with reviewer 1's comment, we have justified the main trial's sample size by stating that a sample size of 40 per arm should be sufficient to avoid under or overpowering the main study whilst not making the pilot study excessively large (Whitehead et al., 2016).*

Reference:

Whitehead AL, Julious SA, Cooper CL, Campbell MJ. Estimating the sample size for a pilot randomised trial to minimise the overall trial sample size for the external pilot and main trial for a continuous outcome variable. Statistical Methods in Medical Research. 2016;25(3):1057–73 Available from: https://pubmed.ncbi.nlm.nih.gov/26092476/

- P 8, lines 3-5: This is a very interesting aim in your study: "This will be achieved by seeking to understand the causes of the behaviour change data obtained from the trial." I think it would be even more interesting if you also tried to understand the reasons for the lack of behaviour change (since we know that a significant portion of patients do not improve after depression treatment).

- *In line with reviewer 1's comment, we have edited this sentence to read: "this will be achieved by seeking to understand the potential relationships between factors obtained from the trial data" (page 8), as this fits more with what a process evaluation attempts to evaluate.*

Methods

- Again, this is perhaps beyond the scope of this protocol, so please excuse me if so. But I would like to see a rationale for choosing the waitlist as your control group. It is sometimes questionable to use a control group that is less effective than existing treatments, particularly from an ethical standpoint. Please refer to Gold et al. (2017), which state that a comparison group needs to be safe, potentially effective, and desirable.

Gold, S. M., Enck, P., Hasselmann, H., Friede, T., Hegerl, U., Mohr, D. C., & Otte, C. (2017). Control conditions for randomised trials of behavioural interventions in psychiatry: a decision framework. *The Lancet Psychiatry, 4*(9), 725-732.

- *Thank you for this comment. This raises an interesting point; the reason a waitlist group (i.e. treatment as usual) was chosen is that we have two active groups (i.e., supported and unsupported arms) thus we are not only comparing to the waitlist group. We agree that it would have been more desirable for the waitlist group to receive SPARX, however our Sponsor who provides our indemnity felt that it would not be ethical to offer a digital health intervention that has not been medically approved in the UK to those on a waitlist (who are not being actively monitored). Nonetheless, previous research with SPARX has shown that the extra support given to young people as part of being in a study can be beneficial, as in the Dutch study of SPARX (Poppelaars et al., 2016) whereby those in the monitored controlled group also saw improvements. We have now added this rationale on page 7.*

- P 10, lines 40-44: add a reference already in the first sentence where thematic analysis is mentioned: "As the process evaluation is a combination of exploration and description, thematic analysis will be used to identify, analyse, and report patterns within the transcribed interviews (REF)."

- *We thank the reviewer for pointing this out and have added the following reference on page 15.*

Reference:

Braun V, Clarke V. Successful Qualitative Research: A Practical Guide for Beginners. SAGE Publications; 2013.

- P 12, lines 40-41. I don't disagree with you, but could you please elaborate on what you mean when you say that thematic analysis is regarded as the most flexible qualitative analytical process?

- *We have now clarified in the manuscript on page 16 that: "by this, we mean that thematic analysis can be applied across methodologies and epistemologies (Braun & Clarke, 2012)."*

Reference:

Braun, V., & Clarke, V. (2012). Thematic analysis. APA Handbook of Research Methods in Psychology, Vol 2: Research Designs: Quantitative, Qualitative, Neuropsychological, and Biological., 2, 57–71. https://doi.org/10.1037/13620-004

- You plan independent chi-squared tests and between-group t-tests. However, let's consider if you have enough power to compare groups accurately. This usually requires larger sizes per group when evaluating depression treatment (due to spontaneous remission and moderate effectiveness in general). A rough estimate is that around 75 to 100 participants per each group. It might be a good idea to stick with within-group t-tests for now and keep those between-group comparisons in mind for a future, larger-scale trial. I'd love to hear your thoughts if you see things differently.

- *Thank you for your insightful comment regarding the statistical approach and sample size considerations. We acknowledge that between-group comparisons require sufficient power, particularly in trials evaluating depression interventions where spontaneous remission and moderate effect sizes can complicate detection of significant differences. Our current feasibility study is not powered for definitive between-group comparisons of efficacy but aims primarily to assess feasibility, acceptability, and mechanisms of engagement. We have now added this to manuscript on page 16 to make this clear to the reader.*
- *Given this, we agree that within-group analyses (e.g., paired t-tests) may be more appropriate for evaluating pre-post changes within conditions, rather than between-group comparisons, which would be more suitable for a future, fully powered trial. However, we believe it is still valuable to conduct exploratory between-group comparisons (e.g., independent t-tests, chi-squared tests) for process evaluation data with appropriate caution, acknowledging the limitations in statistical power. Besides, process evaluations are often exploratory in nature thus not requiring large sample sizes.*

- P 14, lines 7-8. Please specify the contextual variables you will examine using multiple linear regression models. A brief mention in parentheses would suffice.

- *In line with reviewer 1's comment, we have now added the contextual variables we will examine on page 16*

- P 15, lines 9-10. Kindly include your plan for disseminating the results of this study.

- *We have now added our dissemination plan for the study findings on page 17.*

- Methods: I would like to see some details on participant recruitment for the trial. Please include this or direct me to where I can find it. Under "Sampling and recruitment for adolescent and parent/guardian interviews," I found recruitment plans for the qualitative interviews but not for the trial itself. Perhaps this again because this information belongs to the trial protocol?

- *The reviewer is correct in stating that this information belongs in the trial protocol and not the process evaluation protocol. We had been mindful of re-stating trial details due to word limits, however we have added a sentence to the methods section to direct readers to the trial protocol: "please see the published protocol paper for study information related to the trial (Khan et al., 2024).*

- I also miss a description of how you plan to manage safety in this study (how to assess and monitor suicidality, how to monitor and report adverse events, etc.). Perhaps this was left out intentionally since it is a protocol for the process evaluation. If so, could you refer to your full study protocol and add this to the supplements, maybe? study protocol (18) (26/03/2024).

- *Indeed, this was omitted as the information is in the trial protocol, however, we have also added that: "full details of consent procedures and how we will manage safety (i.e. monitoring suicidality and monitoring and reporting adverse events) are described in our published protocol paper" under 'Ethics and dissemination' on pages 7.*

Minor comment

- Page 5, line 5-6 : Should it be characterised rather than categorised? I believe also add "a" before persistent mood.

We thank the reviewer and have edited the manuscript accordingly on page 4.

VERSION 2 - REVIEW

**Reviewer**	**1**
**Name**	**Richards, David**
**Affiliation**	**University of Exeter, Institute for Health Research**
**Date**	**24-Jun-2025**
**COI**	

Thank you for your comprehensive editing of your paper. I think you have addressed my comments well and the logic model and conceptual red threads through the paper are clear now. One minor quibble is that you still revert to the third person from time to time, particularly in the abstract. For consistency's sake I would have a close read and amend where you find yourself straying from the first person voice. Other than that I have no further comments to make.
